# C-Terminal Binding Protein: Regulator between Viral Infection and Tumorigenesis

**DOI:** 10.3390/v16060988

**Published:** 2024-06-19

**Authors:** Meihui Huang, Yucong Li, Yuxiao Li, Shuiping Liu

**Affiliations:** 1 Xiangya School of Medicine, Central South University, Changsha 410013, China; 8303221328@csu.edu.cn (M.H.); lyc0680@csu.edu.cn (Y.L.); 8304220215@csu.edu.cn (Y.L.); 2 Department of Microbiology, School of Basic Medical Science, Central South University, Changsha 410013, China

**Keywords:** C-terminal binding protein (CtBP), transcriptional coregulator, corepressor, viral infection, tumorigenesis

## Abstract

C-terminal binding protein (CtBP), a transcriptional co-repressor, significantly influences cellular signaling, impacting various biological processes including cell proliferation, differentiation, apoptosis, and immune responses. The CtBP family comprises two highly conserved proteins, CtBP1 and CtBP2, which have been shown to play critical roles in both tumorigenesis and the regulation of viral infections. Elevated CtBP expression is noted in various tumor tissues, promoting tumorigenesis, invasiveness, and metastasis through multiple pathways. Additionally, CtBP’s role in viral infections varies, exhibiting differing or even opposing effects depending on the virus. This review synthesizes the advances in CtBP’s function research in viral infections and virus-associated tumorigenesis, offering new insights into potential antiviral and anticancer strategies.

## 1. Introduction

C-terminal binding protein (CtBP), identified by Boyd et al. during research on the human adenovirus type 5 E1A gene, is a transcriptional co-repressor that binds to the C-terminal region of the E1A protein [1]. Alternative RNA splicing and post-translational modification generate multiple CtBP isoforms with distinct temporal and spatial expression patterns, suggesting isoform-specific functions. The CtBP family controls cellular processes by acting as transcriptional corepressors and cytoskeletal regulators [2]. Overexpression of CtBP is often associated with processes such as epithelial-mesenchymal transition (EMT) and self-renewal of tumor stem cells [3,4], which promotes the development and progression of a variety of tumors, such as hepatocellular carcinoma, prostate cancer, gastric cancer, breast cancer, etc. Vertebrates possess two homologous CtBP genes, which encode distinct proteins: CtBP1 and CtBP2. These proteins are frequently overexpressed in cancers and function as oncogenic transcriptional coregulators, which may promote cancer development and progression, leading to tumor invasion, which is associated with poor prognosis [5]. Recent research suggests that CtBP regulates the replication of multiple viruses and contributes to the development of virus-associated tumors. This review aims to examine the role of CtBP in viral replication and virus-associated tumorigenesis, with the goal of identifying novel targets and strategies for preventing and treating viral diseases and virus-associated tumors.

## 2. Overview of CtBP

The CtBP family represents a group of evolutionarily conserved transcriptional corepressors that are indispensable for normal development in vertebrates and non-vertebrates. These proteins collaborate with DNA-binding transcription factors to regulate the expression of target genes, thereby influencing various cellular biological processes [1]. CtBPs are abundant in, but not restricted to, the nucleus, where they function as transcriptional co-repressors, whereas in the cytoplasm they control membrane translocation [6]. As transcriptional corepressors, it has been extensively demonstrated that CtBPs interact with diverse transcription factors to form transcriptional complexes, recruiting a variety of chromatin-modifying enzymes and ultimately leading to the repression of gene transcription associated with embryonic development, cell differentiation and division, and tumorigenesis [6,7,8,9,10]. Early studies demonstrated that CtBP can modulate cellular senescence and regeneration in primary fibroblasts by suppressing the transcription of tumor suppressors [11]. It was further established that CtBP acts as a negative regulator of tumor suppressors p16INK4A, E-cadherin, phosphatase, and tensin homolog (PTEN) [11,12], while also serving as a downstream target of tumor suppressors INK4A/Arf, APC, and HIPK2 [13,14], leading to cell apoptosis. The CtBP family encompasses multiple isoforms due to alternative RNA splicing and post-translational modifications [15]. These isoforms exhibit distinct temporal and spatial expression patterns, suggesting functional specificity among them [16].

**Figure 1:**
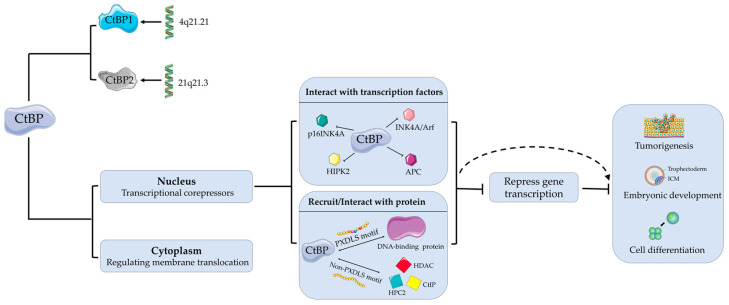
Two types of CtBP and their possible biological roles. CtBP, C-terminal binding protein; p16INK4A, a tumor suppressor protein; INK4A/Arf, Inhibitor of Cyclin-Dependent Kinase 4/alternative reading frame; HIPK2, Homeodomain interacting protein kinase 2; APC, Adenomatous polyposis coli; PXDLS, Pro-X-Asp-Leu-Ser (PXDLS) motif; HDAC, Histone deacetylases; HPC2, Human Polycomb Protein 2; CtIP, CtBP-interacting protein. The CtBP family includes two crucial proteins, CtBP1/CtBP2. CtBP acts as a negative regulator of tumor suppressors p16INK4A, INK4A/Arf, APC, and HIPK2, leading to cell apoptosis. CtBP proteins can exert their functions by recognizing the PXDLS motif within DNA-binding proteins. As for non-PXDLS-containing proteins, CtBP proteins interact with HDAC, HPC2, and CtIP. These interactions ultimately lead to the repression of gene transcription associated with embryonic development, cell differentiation, and tumorigenesis.

CtBPs exert their functions by recognizing the Pro-X-Asp-Leu-Ser (PXDLS) motif within DNA-binding proteins. They can dimerize and potentially influence gene expression by connecting his-tone deacetylases (HDACs) to DNA-binding factors [9]. CtBP proteins interact not only with PXDLS-containing proteins but also with those lacking this motif, such as HDAC1, HDAC2, and HDAC5 [8]. Additionally, they can not only interact with the polycomb protein hPc2 and form a complex with CtIP (CtBP-interacting protein) [17] but also play a role in Golgi apparatus maintenance [18]. One CtBP family member, rCtBP1/BARS50 (brefeldin A ADP-ribosylation substrate 50), has been shown to possess acyltransferase activity, contributing to Golgi apparatus formation and maintenance [6,19]. Furthermore, CtBP dysfunction has been implicated in altered cell adhesion and neurodegenerative diseases [20,21], among others (Figure 1).

CtBP1/BARS acts both as an oncogenic transcriptional co-repressor and as a fission-inducing protein required for membrane trafficking and Golgi complex partitioning during mitosis, hence for mitotic entry. Filograna et al. investigated N-(3,4-dichlorophenyl)-4-{[(4-nitrophenyl)carbamoyl]amino}benzenesulfonamide, a small molecule inhibitor that disrupts the CtBP1/BARS Rossmann fold, thereby inhibiting both its pro-tumorigenic transcriptional activity and its role in mitotic entry [6]. Considering CtBP’s association with the transcriptional corepressor and its self-association, Dcona et al. developed a family of CtBP dehydrogenase inhibitors based on the parent 2-hydroxyimino-3-phenylpropanoic acid (HIPP). These inhibitors specifically disrupt cancer cell viability, abrogate CtBP’s transcriptional function, and block polyp formation in a mouse model of intestinal polyposis that depends on CtBP’s oncogenic functions [22]. Another study also suggests that substrate-competitive inhibition of CtBP dehydrogenase activity is a potential mechanism to reactivate tumor-suppressor gene expression as a therapeutic strategy for cancer [23]. Moreover, CtBP 1 and 2 possess regulatory disomer-specific 2-hydroxyacid dehydrogenase (D2-HDH) domains that provide an attractive target for small molecule intervention. Crystal structures of CtBP 1 and 2 revealed that MTOB binds in an active site containing a dominant tryptophan and a hydrophilic cavity, neither of which are present in other D2-HDH family members [24]. However, ITC experiments show that HIPP binds to CtBP with an affinity 1000-fold greater than that of MTOB, making it a more promising candidate for selective CtBP1/BARS inhibition [24]. In conclusion, while CtBP has emerged as a promising target for cancer therapy, its potential for antiviral applications remains largely unexplored. Existing antiviral treatments do not appear to directly target CtBP, but our review suggests several promising cellular pathways associated with CtBP that could be exploited for novel therapeutic development. Focusing on these pathways holds potential for the creation of effective antiviral drugs.

Given CtBP’s extensive regulatory roles, it is crucial to explore the specific functions of different CtBP isoforms and their interactions with chromatin-modifying enzymes. Future studies should focus on the development of isoform-specific inhibitors to target CtBP-related pathways selectively.

### 2.1. CtBP1

CtBP1, a crucial member of the CtBPs family, is encoded by a gene located on human chromosome 4q21.21 [25]. This protein interacts with a variety of DNA-binding repressors and is involved in the regulation of gene expression. Srinivasan et al. found that the mammalian polycistronic ribosomal (PcG) protein YY1 binds to polycistronic ribosomal response elements in Drosophila embryos and recruits other PcG proteins to DNA. However, in CtBP mutants, recruitment of PcG proteins and corresponding histone modifications does not occur. This study demonstrates that CtBP is required for YY1 DNA binding and PcG recruitment [26]. Three years later, Chinadurai discovered that CtBP mediates coordinated histone modification by deacetylation and methylation of histone H3-Lysine 9 and demethylation of histone H3-Lysine 4 [27]. CtBP also recruits the small ubiquitin-like modifier (SUMO) conjugating E2 enzyme UBC9 and the SUMO E3 ligase (HPC2), which regulates the SUMOylation of transcription factors. It has also been shown that CtBP1 antagonizes the activity of the global transcriptional coactivator p300/CBP [27]. Some other research shows that CtBP1 also mediates the recruitment of chromatin-modifying enzymes, such as HDACs and histone methyltransferases (HMTases), to specific gene promoter regions, thereby repressing gene transcription [28]. As mentioned above, the activity of CtBP1 is modulated by various factors such as phosphorylation [29], SUMOylation [30], and NADH binding [31].

Research has demonstrated that CtBP1 is overexpressed in a wide range of cancers and is associated with increased tumor invasiveness and metastasis, playing a role in cancer development and progression through multiple mechanisms [32,33]. Interestingly, CtBP has also been shown to play an important role in mouse neurodevelopment. The experimental data of Deng et al. showed that CtBP1 directly binds to and transcriptionally represses the promoters of melanoma cell-associated genes (pyruvate carrier 1 and 2 genes, MPC1 and MPC2), leading to an increase in the level of free NADH in the cell membrane and nucleus, and promoting the proliferation and migration of melanoma cells [34,35]. Thus, CtBP1 is not only at the center of tumor metabolism and transcriptional control but also participates in a series of biochemical reactions by regulating intracellular NADH content. Hamada et al. found that CtBP1 expression was detected in the nuclei and cytoplasm of Purkinje cells, in the nuclei of granule cells and molecular layer (ML) cells, and in granule cell axons [36].

Targeting CtBP1’s interaction with chromatin-modifying enzymes presents a promising therapeutic strategy. Future research should focus on developing small molecules or peptides that disrupt CtBP1’s binding to its partners, thereby inhibiting its oncogenic activity.

### 2.2. CtBP2

CtBP2, another critical member of the CtBP family, is encoded by a gene located on chromosome 21q21.3 [25]. It harbors a highly conserved functional region, the unique N-terminal domain (NTR), which comprises 20 amino acids essential for its localization and activity, locates it primarily in the nucleus, and enables it to specifically recognize and interact with proteins containing the PXDLS motif [37]. The C-terminus of CtBP2 forms a distinct domain with NAD^+^ and NADH binding sites, facilitating the dimerization of CtBP monomers into homodimers or heterodimers [38]. NAD(H) promotes tetrameric assembly of human CtBP2, and mutants with an unstable CtBP2 tetramer are defective in oncogenic activity [39].

In addition to being associated with normal cell carcinogenesis, CtBP2 has also been implicated in embryonic development, adipogenesis, and angiogenesis [38,40]. For example, Li et al. showed that CtBP2 promotes high glucose-induced cell proliferation, angiogenesis, and cell adhesion through the Akt signaling pathway, and that CtBP2 may be a potential target for diabetic retinopathy (DR) prevention [41]. Wang et al. used Co-IP to show that CtBP2 interacts with receptor-interacting protein 140 (RIP140). They subsequently found that inflammation, apoptosis, and permeability levels were significantly elevated in CtBP2-overexpressing pulmonary microvascular endothelial cells (PMVECs) in a lipopolysaccharide (LPS)-induced acute lung injury model, but when RIP140 was silenced, these levels were suppressed. Thus, it is suggested that CtBP2 overexpression reversed the inhibitory effect of RIP140 silencing on LPS-induced inflammation, apoptosis, and permeability levels in HPMECs [42]. Sekiya et al. found that CtBP2 also has an important correlation between cellular metabolic levels and the pathogenesis of obesity in humans. Their results showed that CtBP2 can monitor cellular redox status and maintain coordinated metabolic homeostasis, and that the dysfunction of CtBP2 may be a key point in the development of obesity [43].

CtBP2’s involvement in various physiological processes and its overexpression in cancers make it an attractive therapeutic target. Future research should investigate the development of CtBP2-specific inhibitors and their potential in cancer therapy.

## 3. CtBP and Tumors

In recent years, numerous studies have found that CtBP is closely related to tumors and is highly expressed in breast cancer [44], lung cancer [45], ovarian cancer [46], pancreatic cancer [47], esophageal cancer [48], prostate cancer [49], and other tumors (Table 1). CtBP is involved in various processes such as the Warburg effect of tumor cells, epithelial-mesenchymal transition (EMT), and self-renewal of tumor stem cells, which promotes tumorigenesis and development [50].

In addition, CtBP is a target of several tumor suppressors, including the p14/p19ARF tumor suppressor [51,52,53]. It has also been found that 4-methylthio-2-oxobutyric acid (MtoB), a substrate of CtBP, can act as an inhibitor of CtBP at high concentrations and produce toxicity to cancer cells. In a human colon cancer cell xenograft model, MtoB treatment reduced tumor burden and induced apoptosis [40]. Moreover, as demonstrated by Li *et al*., high levels of CtBP expression promoted the therapeutic efficacy of cisplatin. The dimerization status of CtBP is a potential marker for predicting the sensitivity of ovarian cancer patients to platinum-based drugs and is also a target for improving the therapeutic efficacy of platinum-based drugs in ovarian cancer [54]. In addition, CtBP and its negative regulator ARF were negatively correlated in resected human colon adenocarcinoma [53]. CtBP2 interacts with transcription factor 4 (TCF-4) to activate its transcriptional activity causing activation of the downstream target gene β-catenin, which leads to cell migration [55]. In addition, recently Wu et al. found that Snail (Sna) acts as a transcription factor involved in EMT and tumor invasion. The deletion of CtBP inhibits Ras/Sna-induced tumor invasion and Sna-mediated invasive cell migration. Their data pointed out that CtBP and Sna may form a transcriptional complex that regulates JNK-dependent tumor invasion and cell migration in vivo [56], suggesting that CtBP may be a useful therapeutic target for human malignancies [44,57,58,59,60].

**Table 1:** The correlation between CtBP and tumors and possible mechanisms of action have been demonstrated.

Name of Tumor	Role of CtBP
Hepatocellular carcinoma	Involving in constituting a transcriptional repression complex to repress E-cadherin [57]
Abdominal aortic aneurysm	Modulating inflammatory responses and disrupting the matrix [58]
Prostate cancer	Regulating cell proliferation through the c-Myc signaling pathway [59]
Gastric cancer	The expression of CtBP2 is inversely correlated with the disease-free progression of gastric cancer [60]
Breast cancer	Inhibiting intracellular cholesterol abundance, thus increasing EMT and cell migration [44]

Hepatocellular carcinoma (HCC) is one of the human malignant tumors with high morbidity and mortality [61]. HCC has a significantly increased expression of CtBP2 compared with adjacent normal liver tissues [62]. CtBP2 overexpression may be closely related to HCC development and progression, providing new clues for HCC diagnosis and treatment. In HCC cells, p19Arf binds to CtBP1 to inhibit cancer cell infiltration [51]. Studies have found that the transcriptional co-repressor CtBP1 may be a key factor in the transcriptional repression complex involved in the repression of E-cadherin expression [57]. These results showed that CtBP correlated with HCC grade and could be used as a prognostic factor for tumors.

Abdominal aortic aneurysm (AAA) is a life-threatening disease that occurs in the aorta with a potential risk of rupture [63]. A cohort study by Bai et al. found that the expression levels of CtBP1 and CtBP2 were significantly increased in the AAA mouse model. Microarray analysis of AAA-aorta showed elevated expression of five MMP genes (MMP1a, 3, 7, 9, and 12) and three proinflammatory cytokine genes (IL-1β, IL-6, and TNF-α) [58]. Two CtBP-specific inhibitors, NSC95397 and Hipp, inhibited the labile activity of the CtBP in vivo and in vitro and reduced the expression of the MMPs and three proinflammatory cytokine gene expressions [22,64]. This suggests the potential pharmacotherapeutic utility of CtBP inhibitors for AAA in modulating inflammatory responses and disrupting the matrix.

Prostate cancer is the most common malignant tumor of the male genitourinary system, and also the second most common type of malignant tumor in men, after lung cancer [65]. Recent studies have found that CtBP1 is highly expressed and mislocalized in metastatic prostate cancer, and reducing the expression level of CtBP1 can significantly inhibit prostate cancer proliferation and metastasis in vivo and in vitro [66]. It was also found that the expression level of CtBP2 in prostate cancer tissues was higher than in normal tissues, which was closely associated with the malignant behavior of the tumor, indicated by elevated serum prostatic specific antigen (PSA) levels, advanced tumor stage (T3), high Gleason score, and positive extraprostatic extension [67]. Inhibition of CtBP2 decreased the level of c-Myc protein, as well as that of HSPC111, a direct transcriptional target of c-Myc [59]. Another study found that antimony promotes the proliferation of prostate cancer cells by activating the CtBP2-ROCK1 signaling pathway and enhancing the stability of the c-Myc protein. Specifically, CtBP2 transcriptionally regulates the expression of RhoC, a member of the RhoGTPase family, which results in enhanced kinase activity of ROCK1 and promotes the stability of the oncogene c-Myc [68]. The above two results showed that CtBP2 can inhibit the development of prostate cancer through the c-Myc signaling pathway, implying that CtBP2 may have potential as a promising therapeutic approach for the treatment of prostate cancer.

Gastric cancer, a prevalent malignant tumor of the digestive tract, is one of the most common cancers worldwide and is a common cause of cancer deaths [69]. The RB Binding Protein 8 (RBBP8) has been reported to be involved in DNA double-strand break (DSB) repair in various cancers. However, its specific functions and related mechanisms in gastric carcinogenesis have not been systematically studied. Yu et al. discovered that RBBP8 plays a role in chromatin modification by suppressing the histone acetylation level of the P21 promoter through the recruitment of the CtBP co-repressor complex to the BRCA1 binding site [70]. Another study pointed out that the expression of CtBP2 is inversely correlated with the disease-free progression of gastric cancer, indicating that CtBP2 plays a significant role in the progression of gastric cancer. In MAP3K8 (mitogen-activated protein kinase kinase kinase 8)-suppressed EBVaGC (Epstein–Barr virus-associated gastric carcinoma) cells, the expression of CtBP2 is significantly reduced. This suggests that MAP3K8 may influence gastric cancer progression by regulating the expression of CtBP2 [60]. Moreover, drug resistance is a critical factor affecting the treatment of gastric cancer. The resistance of gastric cancer cells to anticancer drugs, such as cisplatin (DDP), remains a significant challenge to patient recovery. Wu et al. found that CtBP1 may enhance DDP resistance in gastric cancer cells by activating RAD51 expression, suggesting that CtBP1 knockdown could provide a novel therapeutic approach for the clinical treatment of gastric cancer patients [71].

As a common malignant tumor worldwide, breast cancer is an important cause of mortality [72]. It was found that CtBP regulates cholesterol homeostasis, mainly by forming a complex with ZEB1 and inhibiting SREBF2 transcription. Moreover, CtBP inhibits intracellular cholesterol abundance, leading to increased EMT and cell migration, and cholesterol negatively regulates the stability of the TGF-β receptor on the cell membrane. They also found that the ability of TGF-β to reduce intracellular cholesterol was dependent on increased recruitment of the SREBF2 promoter by the ZEB1 and CtBP complex. It was ultimately concluded that high expression of CtBP and low expression of SREBF2 and HMGCR were significantly associated with high EMT capacity of the primary tumors and that elevated levels of CtBP in patients’ tumors predicted shorter median survival [44].

In summary, CtBP plays an important role in the occurrence and development of cancer, and the study of CtBP function and regulatory mechanisms is of great significance for cancer treatment and prevention.

## 4. CtBP and Viruses

As mentioned earlier, CtBP is a highly conserved transcriptional co-repressor molecule. It was first identified by its interaction with the C-terminus of the adenovirus E1A protein, hence the name carboxy-terminal binding protein [1]. Researchers found that all transcriptional regulators that bind to CtBP possess the motif PXDLS [73], suggesting that a variety of proteins containing this motif, such as EBNA3C, a protein expressed by the Epstein–Barr virus, may interact with CtBP [74]. However, for viruses such as HIV, although direct interactions between CtBP and the virus have not been particularly emphasized, a role for CtBP in influencing the biochemical processes of these infected cells is evident. General roles in cellular transcription and metabolism suggest that CtBP may influence viral replication and cellular transformation processes.

### 4.1. Adenovirus

Adenovirus type 2/5 is a DNA virus associated with tumorigenesis that primarily infects terminally differentiated epithelial cells of the upper respiratory tract. Due to low cytokinesis activity, adenoviral infection induces cells to synthesize the raw materials necessary for viral replication by expressing specific proteins [75]. E1A is the first viral protein expressed in adenovirus replication, which consists of two major isoforms encoded by two differently spliced mRNAs. Of these, the 12S mRNA encodes the small E1A protein of 243 residues (243R) and the 13S mRNA encodes the large E1A protein of 289 residues (289R) [76,77]. Both proteins are highly conserved throughout evolution and contain two exons sharing the conserved region 1 (CR1), CR2, and CR4, with the 289R protein also having a CR3 structural domain consisting of 46 amino acids [77,78,79].

The N-terminus of the E1A protein is essential in the transformation of baby rat kidney (BRK) cells. However, the C-terminal encoded proteins appear to be dispensable of transforming activity [80]. However, according to some studies, the C-terminus can inhibit N-terminal oncogenic activity [79]. Schaeper U et al. found that a short C-terminal sequence of E1A governs the oncogenesis-restraining activity of exon 2 [81]. Kuppuswamy G et al. used the E1A mutants, which lacked a C-terminus or had a mutation in the C-terminus, to determine its transformation activity. Results showed that these mutants exhibited a hypertransformation phenotype in a synergistic transformation assay with the T24 ras oncogene [82]. Other studies show that CtBP interacts with the C-terminal region of the adenovirus E1A protein and that this interaction is required for effective activation of the E1A response gene, suggesting that E1A may block CtBP-mediated inhibition [83,84]. CtBP is a phosphorylated protein, and its phosphorylation level is regulated during the cell cycle, suggesting that it may play an important role in cell proliferation and immortalization. By studying small deletion mutants within exon 2 of the E1A gene, Boyd et al. found that mutants with residues 225–238 deletion were highly defective in immortalization, implying that the 14-amino-acid region may contain functions important for the negative regulation of tumorigenesis and metastasis. To shed light on this issue, they constructed a chimeric gene encoding a fusion of the C-terminal amino acid of E1A with bacterial glutathione-S-transferase (GST), and analysis of the GST-E1a C-terminal mutant protein showed that the interaction of CtBP with the E1A protein plays a key role in adenoviral replication and oncogenic transformation [1].

The truncated C-terminal sequence of the E1A protein is able to control the tumorigenesis inhibitory activity of exon 2, which binds to the cytosolic phosphoprotein CtBP via the 5 amino acid motif PLDLS (Pro-Leu-Asp-Leu-Ser) [85]. This sequence is more conserved in the E1A protein of human adenovirus. To further understand the mechanism underlying the interaction between E1A and CtBP leads to tumorigenesis suppressor activity, Schaeper et al. searched for additional cellular proteins in complex with CtBP and reported the cloning and characterization of the CtIP protein. The CtIP protein binds to CtBP via the PLDLS motif, whereas the E1A exon 2 peptide containing the PLDLS motif disrupts the CtBP-CtIP complex. The results suggest that the tumorigenesis inhibitory activity of E1A exon 2 may be related to the disruption of the CtBP-CtIP complex through PLDLS motifs [81].

**Figure 2:**
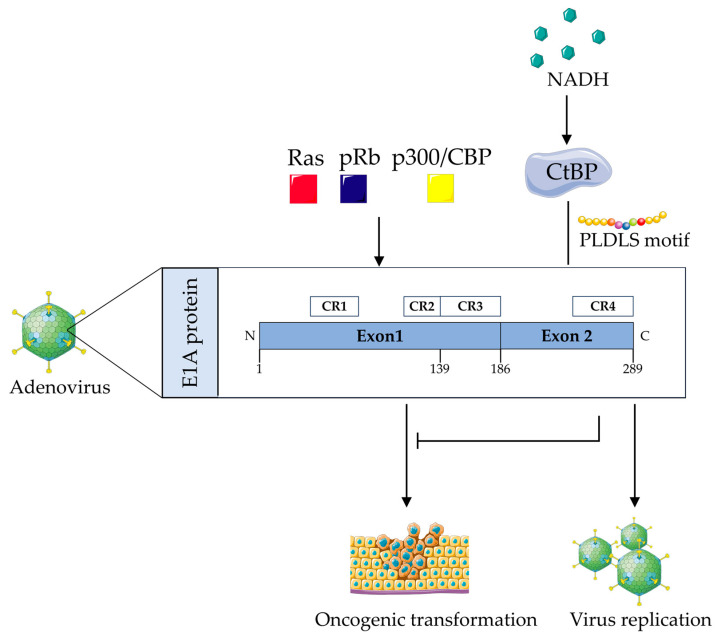
Interaction of CtBP with adenovirus and its role in tumor transformation and viral replication. CtBP, C-terminal binding protein; CR, conserved region; Ras, rat sarcoma; pRB, retinoblastoma; p300/CBP, transcriptional regulators; NADH, nicotinamide adenine dinucleotide; PLDLS motif, Pro-Leu-Asp-Leu-Ser. The adenovirus-expressed E1A protein consists of 289 amino acid residues, of which amino acids 1 to 186 are encoded by exon 1 and amino acids 187 to 289 are encoded by exon 2. On the one hand, transcriptional regulators such as Ras, pRb, and p300/CBP can enhance the tumorigenic-promoting effect of exon 1. On the other hand, exon 2 can inhibit the upstream pathway of exon 1 action, inhibiting cellular immortalization and tumorigenesis, and at the same time, exon 2 also has the biological activity of promoting adenovirus replication. CtBP, as a transcriptional co-repressor protein, can interact with the C-terminal region of the adenovirus E1A protein through the PLDLS motif. During viral infection, intracellular levels of NADH recruit CtBP to affect the activity of the E1A protein and enhance the interaction of CtBP with the E1A protein.

CtBP represses transcription by binding to chromatin-modifying enzymes, such as histone deacetylases and KDM1A H3K4 demethylases [86]. Meanwhile, CtBP is highly homologous to NAD-dependent D2-hydroxyacid dehydrogenases (D2-HDHs), and the experimental results of Balasubramanian et al. demonstrated that NADH induced a conformational change in CtBP, which enhanced the interaction of CtBP with E1A, suggesting that intracellular levels of NADH during viral infections can recruit CtBP to influence the E1A activity [87,88]. When the NADH/NAD^+^ ratio is high in the cell, the NADH-bound dimeric form of CtBP causes global inhibition, thereby maintaining the balance and homeostasis of many cellular processes, under the cell surveillance of p53 and NF-κB. In contrast, in the presence of a low NADH/NAD^+^ ratio, CtBP without NADH monomer blocks p53 function and NF-κB-mediated transcription. In the absence of p53 and NF-kB cellular surveillance, low NADH/NAD^+^ ratios also disrupt homeostatic enzymes and homeostasis, leading to global instability [89]. In conclusion, for adenoviruses, CtBP acts as a co-inhibitor and modifies E1A protein expression, which in turn affects viral replication and immortalization of primary epithelial cells [90] (Figure 2).

Future research should aim to elucidate the precise molecular mechanisms by which CtBP influences adenovirus replication and transformation. This could lead to the development of targeted antiviral therapies that inhibit CtBP-E1A interactions, potentially controlling adenovirus-related diseases.

### 4.2. Epstein–Barr Virus

The Epstein–Barr virus (EBV) is a member of the gammaherpesvirus subfamily [91]. EBV infects resting B-lymphocytes in vitro and drives them to proliferate in the form of a lymphoblastoid cell line (LCL), which has been used as a model to study the development of EBV lymphomas [92,93]. EBNA3C (Epstein–Barr virus nuclear antigen 3C) is one of the virally encoded EBV nuclear antigens known to play multiple roles in viral replication, transformation of infected cells, and immune evasion. EBNA3C can interact with a variety of transcriptional repressor molecules to regulate the transcription of host genes [94]. Earlier studies have shown that EBNA3C interacts with many transcriptional repressors and activators that regulate the transcription of host genes when recruited to EBNA3C binding sites [74], among which CtBP acts as a metabolically induced transcriptional repressor, whose dimerization and repressive activity is dependent on NADH binding [95]. Ohashi M et al. studied how EBV protein EBNA3C promotes B-lymphocyte transformation. However, they found that, contrary to previously proposed models, EBNA3C does not recruit CtBP to the promoters of these genes, and that the interaction of EBNA3C with CtBP is important for both EBNA3C-mediated activation and repression of host genes [93]. Other studies have shown that EBNA3C also interacts with CtBP1 through its C-terminal PLDLS motif and that this interaction appears to be important for some EBNA3C repressions [96].

As previously described, CtBP is a metabolism-sensing transcriptional repressor and can interact with EBNA3C via motif PLDLS. These results suggest that CtBP binds to the promoters of host genes without the presence of EBNA3C and that EBNA3C can interfere with the repressive function of CtBP by interacting with CtBP, leading to EBNA3C-mediated up-regulation of host genes, whereas the interaction of EBNA3C with CtBP may be important for the role of the p300 coactivator in certain EBNA3C-induced recruitment on genes is important [93]. Interestingly, the results of this experiment show that EBNA3C does not appear to promote CtBP recruitment, but rather interferes with CtBP inhibitory activity to up-regulate certain host genes, meaning that interactions with CtBP-repressor proteins are more important for the ability of EBNA3C to induce host gene expression than EBNA3C-mediated repression. These findings reveal an important role for CtBP in the regulation of host gene expression by EBNA3C and a novel mechanism for how EBNA3C activates host genes by interfering with the repressive function of CtBP [74,93,94]. Understanding how EBNA3C disrupts CtBP’s repressive functions offers a potential avenue for therapeutic intervention. Future studies should focus on developing inhibitors that prevent CtBP-EBNA3C interactions, which could help in controlling EBV-associated cancers.

### 4.3. Hepatitis B Virus

Due to the hepatophilic nature of Hepatitis B Virus (HBV), the replication process involves the conversion of HBV incomplete closure of double-stranded DNA into covalently closed circular DNA (cccDNA) after entry into the hepatocyte using the cellular DNA repair reaction to form the viral microchromosome [97]. Under the regulation of HBx, cccDNA transcribes HBV mRNA and pregenomic RNA (pgRNA) [98]. Stochastic integration of HBx genes with the host genome may lead to proto-oncogene activation and tumor suppressor gene inactivation. CtBP binds to HBV and affects metabolism and replicative gene expression in hepatocytes. The CtBP–CtIP (carboxy-terminal binding protein-responsive protein) complex may link the transcriptional repression activity of CtBP to the DNA repair function of CtIP, which can interact with CtBP through a conserved sequence in its N-terminal region. Although the exact mechanism and outcome of this interaction are not fully understood, it has been suggested that this interaction may affect the function of CtIP in DNA repair [99]. Liu et al. examined the mRNA and protein expression levels of CtIp in a HepG2 hepatocellular carcinoma cell line stably expressing HBx, and the results showed that the cell line was blocked in the G2/M phase of the cell cycle, and HBx down-regulated both CtIP protein expression level and mRNA expression level [100]. Therefore, HBx can interfere with the expression of the tumor suppressor protein CtIp and alter the activity of the CtBP-CtIP complex, which in turn affects cellular DNA transcription activity and damage repair. Investigating the role of CtBP in HBV replication and its interactions with host proteins could reveal novel therapeutic targets. Developing small molecule inhibitors that disrupt CtBP-HBx interactions may provide a new approach to treating HBV infections and preventing liver cancer.

### 4.4. Human Immunodeficiency Virus

Human immunodeficiency virus (HIV) can invade the human brain during the early stages of infection [101]. However, its interactions with the blood–brain barrier (BBB) cells remain poorly studied [102]. Victor et al. found that CtBP is closely associated with HIV transcription. CtBP1 plays an important role in HIV-infected pericentromeric cells and is strongly associated with occludin levels and SIRT-1 activity. During the first 48 h of HIV infection, intranuclear translocation of CtBP1 increases with the improvement of HIV transcriptional efficiency. CtBP1 is a transcriptional co-repressor and NF-κB is a key regulator of HIV transcription. The NADH-dependent intranuclear translocation of CtBP1 is associated with reduced activity of SIRT-1 (deacetylase), which normally acts as a transcriptional co-repressor by deacetylating and inhibiting NF-κB activity. During early HIV invasion of the brain and interaction with the blood–brain barrier, there is a regulatory role for CtBP1 nuclear translocation by the brain pericyte tight junction protein occludin, with occludin levels decreasing by approximately 10% within 48h post-infection. Reduced levels of occludin correlate with an increase in the nuclear translocation of CtBP1. This leads to an increase in NADH levels, which in turn reduces NAD^+^-dependent intranuclear translocation of CtBP1, decreases SIRT-1 activity, and increases NF-κB acetylation and HIV transcription. SIRT-1 expression and phosphorylation levels were restored after the recovery of occludin levels [103]. In pericytes with occludin overexpression, cytoplasmic levels of CtBP1 were increased and nuclear localization was reduced, suggesting that changes in the level of subcellular localization of CtBP1 allow HIV transcription to be affected. Targeting the regulatory role of CtBP1 in HIV transcription could lead to novel antiviral strategies. Future research should explore the development of therapies that modulate CtBP1’s nuclear translocation and interaction with host proteins, potentially reducing HIV replication and its effects on the central nervous system.

### 4.5. Marek’s Disease Virus

For Marek’s disease virus (MDV), there is an interaction between the encoded protein MEQ (MDV EcoRI Q fragment) and CtBP1, which has been implicated in MDV-induced tumor formation [104]. MEQ is the major viral oncogenic protein of MDV. It was found that MEQ can interact with CtBP through the PLDLS motif, and this interaction is critical for MDV oncogenicity. MDV mutants defective in CtBP interaction, constructed by reverse genetics techniques, showed a complete loss of oncogenicity in experiments but were still able to replicate. Thus, non-oncogenic MDV generated by mutating the CtBP interaction domain of MEQ has the potential to be an improved vaccine against pathogenic MDV infection [105]. Zhao et al. analyzed the results by confocal microscopy and showed that MDV infection induced nuclear accumulation of Hsp70 and its co-localization with Meq. Subsequently, they confirmed the Meq-Hsp70 interaction using bidirectional immunoprecipitation using Meq- and Hsp70-specific antibodies [106]. This suggests that the interaction between Meq and Hsp70 is important in the promotion of MDV tumorigenesis by CtBP. Developing CtBP inhibitors that prevent its interaction with MEQ could serve as an effective strategy for controlling MDV infections. Such inhibitors may also have potential as improved vaccines against pathogenic MDV strains.

### 4.6. Zaire Ebola Virus

CtBP can interact with BARS to influence cellular signal transduction and functional regulation [6]. CtBP/BARS regulates macroendocytosis by phosphorylating LIMK and CtBP/BARS proteins to promote actin remodeling and macroendosome closure [107]. It is shown that the Zaire Ebola virus (ZEBOV) utilizes a mechanism similar to macroendocytosis to achieve endocytosis by promoting local actin polymerization and membrane perturbation to invade host cells. This endocytosis is not dependent on clathrin, caveolae, or dynamin, but requires cholesterol-rich lipid rafts. CtBP/BARS plays an important role in ZEBOV (Zaire ebolavirus) viral infections. The CtBP/BARS protein is thought to be a factor that replaces dynamin in macropinocytosis, and it promotes the disconnection of nascent giant vesicles from the plasma membrane. It was found that inhibition of CtBP/BARS expression using siRNA significantly reduced ZEBOV infection, suggesting that CtBP/BARS plays an important role in ZEBOV entry into host cells and is a key regulator of macropinocytosis [6,108]. By inhibiting the function of CtBP/BARS, ZEBOV infection can be significantly reduced, providing a potential target for future therapeutic options. Targeting CtBP/BARS-mediated pathways could provide a novel therapeutic approach for Ebola virus infections. Future research should focus on identifying and developing inhibitors that specifically disrupt CtBP/BARS function, potentially reducing the viral load and improving patient outcomes.

## 5. Conclusions and Prospects

In summary, CtBP plays a significant role in a variety of tumorigenesis and viral-related diseases, but the specific pathways of action are unclear and the mechanism of interaction with a variety of cancer molecules or viral antigens is unknown, which makes CtBP play different roles in different tumors or viral replication processes. Meanwhile, the two members of the CtBP family, CtBP1 and CtBP2, have different molecular structures and different binding targets, which ultimately means that they show different physiological activities, even in the same tumor or viral disease. For example, on the one hand, in hepatocellular carcinoma, CtBP1 acts as a transcriptional co-blocker to inhibit cellular expression of E-cadherin, which results in the loss of intercellular adhesion and promotes pathological processes such as epithelial–mesenchymal transition, cancer cell migration and invasion into the stroma. On the other hand, CtBP2 may inhibit the expression level of oncogenes through the formation of transcriptional co-repressors, thereby promoting hepatocellular carcinoma. The reason for this situation may be that CtBP1/2 are regulated by different substances in different tumor microenvironments. However, for adenovirus infection, instead of promoting tumorigenesis, the high expression of CtBP served to inhibit the growth of cancer cells. Moreover, in virus-infected cells, CtBP activity is also virus-specific, including affecting host cell gene expression (EBV, HBV), influencing viral replication (HIV), regulating viral entry into host cells (ZEBOV), etc. The diversity of physiological roles of CtBP may be related to the differences in surface antigens of the viruses and the types of virus-infected host cells.

In this review, we emphasize the mechanism of interaction between CtBP and adenovirus E1A protein. We found that in adenoviruses, CtBP1 and CtBP2 have opposite physiological roles, i.e., exon 1 promotes tumorigenesis, whereas exon 2 acts as a repressor of exon 1, inhibiting cellular immortalization and tumorigenesis, and exon 2 also has the biological activity of promoting adenoviral replication. By comparing the effects of CtBP in different tumors and viruses, we found that CtBP showed completely different biological activities, and sometimes the two conclusions seemed to contradict each other, suggesting that the specific mechanism of CtBP’s effects in different tumors and viruses is still not completely clear, and needs to be further studied in the future.

Given the important role of CtBP in a variety of viral infections and tumorigenesis and development, future research will focus on exploring CtBP in greater depth as a target for clinical disease therapy. This includes exploring the molecular mechanisms by which CtBP interacts with specific viral proteins, the role of CtBP in the formation of the tumor microenvironment, and how these interactions affect tumor progression, viral replication, and host immune responses. Meanwhile, more detailed studies on the mechanism of CtBP action in different types of viral infections and cancers will be carried out in the future to develop and test small molecule inhibitors targeting the CtBP site of action. Ultimately, future work will focus on clinical trials that will advance CtBP inhibitors, evaluate their safety and efficacy in cancer patients, and explore their potential to be used in combination with other therapeutic approaches, facilitating their translation to clinical applications and providing new strategies and approaches for cancer therapy and the treatment of virus-associated tumors.

## Data Availability

Data availability is not applicable to this article as no new data were created or analyzed in this study.
